# Heart-type Fatty acid-binding protein in Acute Myocardial infarction Evaluation (FAME): Background and design of a diagnostic study in primary care

**DOI:** 10.1186/1471-2261-8-8

**Published:** 2008-04-15

**Authors:** Madeleine HE Bruins Slot, Geert JMG van der Heijden, Frans H Rutten, Onno P van der Spoel, E Gijs Mast, Ad C Bredero, Pieter A Doevendans, Jan FC Glatz, Arno W Hoes

**Affiliations:** 1 Julius Center for Health Sciences and Primary Care, University Medical Center Utrecht, PO Box 85500, 3508 GA Utrecht, The Netherlands; 2 St. Antonius Hospital Nieuwegein, Department of Cardiology, 3435 CM Nieuwegein, The Netherlands; 3 Diakonessenhuis Utrecht, Department of Cardiology, 3582 KE Utrecht, The Netherlands; 4 Department of Cardiology Division Heart and Lungs, University Medical Center, 3508 GA Utrecht, The Netherlands; 5 Department of Molecular Genetics, Cardiovascular Research Institute Maastricht (CARIM), Maastricht University, 6200 MD Maastricht, The Netherlands

## Abstract

**Background:**

Currently used biomarkers for cardiac ischemia are elevated in blood plasma after a delay of several hours and therefore unable to detect acute coronary syndrome (ACS) in a very early stage. General practitioners (GPs), however, are often confronted with patients suspected of ACS within hours after onset of complaints. This ongoing study aims to evaluate the added diagnostic value beyond clinical assessment for a rapid bedside test for heart-type fatty-acid binding protein (H-FABP), a biomarker that is detectable as soon as one hour after onset of ischemia.

**Methods:**

Participating GPs perform a blinded H-FABP rapid bedside test (Cardiodetect^®^) in patients with symptoms suggestive of ACS such as chest pain or discomfort at rest. All patients, whether referred to hospital or not, undergo electrocardiography (ECG) and venapunction for a plasma troponin test within 12–36 hours after onset of complaints. A final diagnosis will be established by an expert panel consisting of two cardiologists and one general practitioner (blinded to the H-FABP test result), using all available patient information, also including signs and symptoms. The added diagnostic value of the H-FABP test beyond history taking and physical examination will be determined with receiver operating characteristic curves derived from multivariate regression analysis.

**Conclusion:**

Reasons for presenting the design of our study include the prevention of publication bias and unacknowledged alterations in the study aim, design or data-analysis. To our knowledge this study is the first to assess the diagnostic value of H-FABP *outside *a hospital-setting. Several previous hospital-based studies showed the potential value of H-FABP in diagnosing ACS. Up to now however it is unclear whether these results are equally promising when the test is used in primary care. The first results are expected in the end of 2008.

## Background

For a general practitioner (GP), diagnosing or excluding acute coronary syndrome (ACS; comprising unstable angina (UA) and acute myocardial infarction (AMI)) often poses a diagnostic dilemma. On the one hand, missing an ACS may lead to excess morbidity and mortality that could have been prevented with optimal treatment. Guidelines therefore recommend immediate hospital referral in patients suspected of ACS, even when suspicion is relatively low [1,2]. On the other hand, unjustified referral of patients without ACS increases workload in the emergency department and causes unnecessary anxiety in both patients and their relatives. Consequently, adequate diagnostic assessment, correctly identifying ACS patients, while limiting unnecessary referral of non-ACS patients is desirable, but may be difficult to achieve.

Although some patients with chest pain or other symptoms suggestive of ACS will contact emergency services directly, the majority of patients will consult a GP first. Typically, the GP will assess these patients using history taking and physical examination only. With these limited tools it is notoriously difficult to accurately rule out or rule in ACS, notably in women and elderly patients in whom signs and symptoms of ACS can be rather atypical [3]. An electrocardiogram (ECG) may provide additional diagnostic information in the assessment of ACS, but is often not available in primary care. Moreover, the initial ECG of a patient with AMI does not always reveal ST-segment elevation or Q-wave changes, indicative of infarction [1]. Alternatively, biomarkers of myocardial damage could be useful as these, after their appearance in plasma, show 100% sensitivity. Currently, troponin is the biomarker of choice according to European and American guidelines on myocardial infarction. Unfortunately, troponin is elevated only 6–9 hours after onset of ischemia [1,4,5], while most patients with symptoms suggestive of ACS present themselves to the GP between 1 and 3 hours after symptom onset [6-8]; hours before troponin can be used to accurately exclude or confirm AMI.

Recent studies in laboratories and the emergency department have shown that heart-type fatty acid-binding protein (H-FABP), a more recently developed cardiac biomarker, is able to detect myocardial damage as soon as one hour after onset of ischemia and, therefore, is regarded the earliest plasma marker available [9-11]. A bedside test for H-FABP, providing results within 15 minutes [12], could potentially reduce diagnostic uncertainty for patients suspected of ACS in primary care. We therefore sought to determine the diagnostic accuracy and feasibility of a rapid bedside test for H-FABP in patients suspected of ACS in primary care.

### Objectives

This study aims to assess the diagnostic value of a rapid bedside test for H-FABP, in addition to history taking and physical examination in primary care patients suspected of ACS. In addition, the balance between costs and effects of applying the H-FABP bedside test in primary care will be evaluated.

## Methods

### Study design and data collection

**Figure 1:**
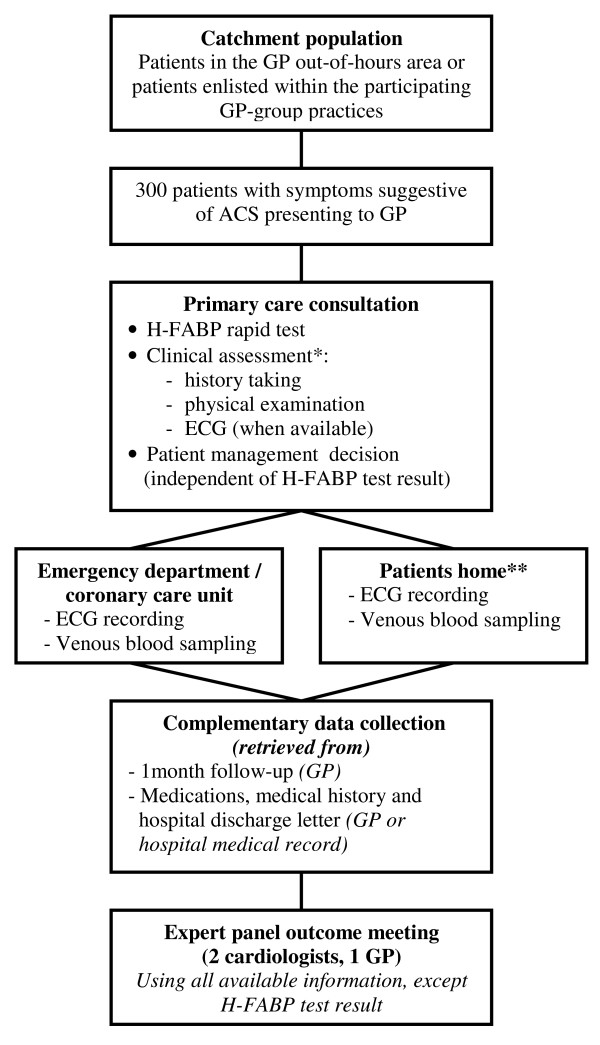
**Flow diagram of study design**. Abbreviations: GP = general practitioner, ACS = acute coronary syndrome, H-FABP = heart-type fatty acid-binding protein, ECG = electrocardiogram. * The clinical score is based on ref. 13. ** Measurements are performed by qualified GP laboratory personnel.

The study design is depicted in figure 1. Patients are primarily recruited by GPs working at one of three participating out-of-hours GP services in the region of Utrecht, The Netherlands (1 urban and 2 semi-urban). Additionally, 25 GPs from group practices will recruit patients during daytime hours. Diagnostic assessment during the initial GP consultation includes standardised history taking and physical examination and rapid H-FABP testing (see below). To allow for a definitive decision whether ACS is present ("gold" or reference standard) an ECG is recorded and a venous blood sample is collected in all patients (for measurement of currently preferred biomarkers including troponin, creatinin kinase (CK) and creatinin kinase-myocardial band (CK-MB)), irrespective of whether or not they are referred to hospital. In patients who are referred to hospital these measurements are performed as part of routine care. Patients who are not referred to hospital are visited at home by qualified GP laboratory service personnel to perform the above mentioned tests. Blood samples are obtained between 12 to 36 hours after onset of complaints in order to allow for a definitive diagnosis of AMI. Using this time interval we adopt a safe margin for a troponin rise to become detectable in the blood.

The study protocol was approved by the Medical Ethics Committee of the University Medical Center Utrecht, The Netherlands.

### In- and exclusion criteria

All patients with symptoms suggestive of ACS who present themselves to a GP are eligible for inclusion in the study. Presenting symptoms will typically include chest pain or discomfort at rest, but also atypical "vague" complaints such as abdominal discomfort, dizziness or sudden onset of dyspnoea.

Excluded are patients with complaints lasting more than 24 hours, as H-FABP levels usually return to normal 24–36 hours after onset of myocardial ischemia [9,11]. Also excluded are patients who require instant hospital referral and those patients in whom no written informed consent is obtained.

### H-FABP test and clinical score

The H-FABP bedside test (Cardiodetect^® ^Rennesens GmbH, Berlin) can easily be performed by the GP by drawing four drops of capillary whole blood from the patient's finger and applying them onto the test-strip. Within 15 minutes the H-FABP test result (elevated or non-elevated plasma FABP) is available. For study purposes the result is concealed by a blinding-strip. The test is de-blinded by the GP after he/she has made the referral decision. Test results are documented on a standardized case record form, together with findings from history taking and physical examination. Other items on the form include age, gender, prior AMI and treatment for AMI (bypass surgery or percutaneous coronary intervention). Also recorded are patient delay and doctor delay and a probability estimate by the GPs (prior to the H-FABP test result) that a patient has ACS, their decision about referral to hospital and the result of the H-FABP test.

A previous study by Grijseels et al provided a pre-hospital decision rule based on items from history taking and physical examination for patients suspected of ACS [13]. We will validate the diagnostic accuracy of this clinical score and estimate the added value of the H-FABP bedside test.

### Outcome

An expert panel consisting of two cardiologists and one GP will establish the final diagnosis using all available patient information, including signs and symptoms, ECG and biomarker levels (troponin-T or -I, CK and CK-MB). These results are available for all patients, including patients that are not referred to hospital, as they are visited at home by qualified GP laboratory personnel for performance of ECG and laboratory tests.

AMI will be defined in accordance with guidelines from the European Society of Cardiology and the American College of Cardiology [1,5] The diagnosis of AMI is established when patients have suggestive symptoms (such as chest pain) and a maximal concentration of troponin T or I exceeding the decision limit (99^th ^percentile of the values for a reference control group) within the first 36 hours after the onset of complaints and/or CK-MB values greater than two times the upper reference limit on at least one occasion during the same time frame. The presence of ST- and T-wave changes on the ECG, notably ST elevations and Q-waves, can further confirm AMI. Unstable angina (UA) is defined as symptoms of chest pain and ST- and/or T-wave changes on the ECG suggestive of ischemia, without elevation of troponin or CK-MB above the decision limits [1,5].

### Patient management

As already mentioned, every GP will decide about referral to hospital in accordance with daily practice, using only history taking and physical examination and, when available, ECG. This decision is made without using the H-FABP test result. For safety reasons an exception is made for patients with a *positive *H-FABP test result in whom the GP initially decided *not *to refer. In these cases, the GP is instructed to change his initial management decision in favor of hospital referral.

### Statistical analyses

Using 2 by 2 tables the diagnostic value of the H-FABP test alone and in combination with the clinical score will be assessed, using AMI as the outcome, and positive and negative predictive values, sensitivity and specificity will be calculated with 95% confidence interval.

Multivariable regression analysis with receiver operating characteristic (ROC) curves will be used to determine whether the H-FABP test provides added diagnostic value beyond history taking and physical examination (summarized in a clinical score). Two diagnostic models will be tested: one using only the clinical score, the other one consisting of the clinical score together with the H-FABP test result. This will lead to 2 different areas under the ROC-curve (AUC), where the difference in AUC presents the added value of the H-FABP test.

As AMI is notoriously difficult to diagnose in women and the elderly we will perform subgroup analyses in these specific patient categories [1].

### Sample size and power calculation

A frequently used 'rule of thumb' recommends that for each diagnostic determinant included in a multivariable logistic regression analysis at least 10 events (in this case AMI) are necessary [15,16]. Our study includes 2 diagnostic determinants (clinical score and H-FABP test result). Thus, a population in which at least 20 patients with AMI is required. Although available estimates vary, Dutch studies show that in more than 10% of patients suspected of AMI by the GP the diagnosis is confirmed [2,13]. Therefore, 200 patients with suspected AMI need to be included in our study. We will include 300 patients to allow for subgroup analyses.

## Design issues

### Blinding

In diagnostic prediction research the physician ideally should be blinded to the results of the test under study in order to prevent bias in the ascertainment of the disease. In this study, the H-FABP test result may influence the inferences drawn from medical history and physical examination, and thereby influence the referral decision of the GP, especially since it is notoriously difficult to decide about the presence or absence of an ACS based on clinical assessment only.

There are however two important reasons why a fully blinded H-FABP test is not feasible in our study. Firstly, previous hospital based studies showed that the H-FABP bedside test has a positive predictive value of well above 80% [17-20]. We therefore instructed the GPs to decide on the referral before de-blinding the test result, but for safety reasons we also instructed them to refer patients with a positive H-FABP test result to hospital irrespective of their initial referral decision. Secondly, in the hours following application of the test, discoloration of the test-strip occurs which negatively influences the interpretation and thereby the accuracy of the test results. Therefore the test has to be read shortly after its performance.

### Informed consent

It is neither very realistic nor feasible to ask written informed consent to study participation from a patient in an acute life threatening situation, such as with symptoms suggestive for ACS. Therefore the Medical Ethics Committee agreed to ask verbal consent from patients for taking the H-FABP test by the GP. Subsequently patients are given the opportunity for written informed consent after having read an information letter at a more convenient moment. Patients may also decide to withdraw their consent then or at any time thereafter. Only patients who return a *written *informed consent are included in our study.

### Recruitment

We have chosen for a phased introduction of our study in 3 different GP out-of-hour services (weekdays from 5 pm-8 am and weekends). Participating centers are notified of the study progress by a monthly overview of the number of participants and a 2-monthly newsletter with background information on the study, frequently asked questions and tips and tricks, for instance on how to draw the required amount of capillary blood. We anticipate including 300 patients within 36 months.

## Preliminary Results

In March 2006 we started enrollment of patients. In September 2007, 172 patients were included; i.e. monthly enrollment of about 12 patients. Conclusion of enrollment is anticipated before the summer of 2008. Baseline characteristics of the first participants are summarized in Table 1.

**Table 1:** Preliminary patient baseline characteristics (N = 172)

	Number (%)
Demographics	
Age (years, mean ± SD)	66 ± 14
≥ 75 years	57 (33)
Female	88 (51)
Risk factors (N = 114)	
Current smoker	21 (22)
Diabetes mellitus	24 (21)
Hypertension	51 (45)
Hyperlipidemia	36 (32)
Prior ischemic heart disease	34 (30)
Presenting symptoms	
Chest pain	157 (91)
Radiation of chest pain	109 (64)
Vagal symptoms*	97 (57)
Patient referred to hospital	126 (73)
Duration of symptoms (hours) **	3.0 (IQR 1.4–7.0)

* Including nausea, sweating, pallor

** From symptom onset until time of testing, IQR = interquartile range

## Discussion

The presentation of the design of our study provides the reader the opportunity to get informed about our study in an early stage. Moreover, publishing the design of a study independently of its results allows for a reflection on the design of a study. It helps to reduce publication bias and unacknowledged alterations in the study aims, the study design or data-analysis during its conduct. Finally, this article can be seen as an announcement of upcoming study results that may have an impact on the guidelines on acute coronary syndrome currently used in primary care.

Our study on H-FABP as a new cardiac biomarker is noteworthy as patients are recruited *outside *a hospital setting. The, few, previous studies on H-FABP have been performed in the emergency room or by ambulance personnel [17-23], representing an entirely different domain of patients than those seen by the GP. This is also illustrated by the difference in prevalence of AMI in these populations. Hospital-based studies reported a prevalence of AMI around or above 50% in those suspected of AMI [17-21,24], with the exception of one study that reported a prevalence of 16% [22]. Studies performed in primary care observed prevalences as low as 5 or 8% [13,25]. By definition this difference in prevalence has an important impact on the added diagnostic value of the novel rapid bedside test.

Many (earlier) diagnostic studies focus on the performance of a single test, ignoring the information obtained from history taking and physical examination. Based on symptoms and signs however, the likelihood of ACS may increase or decrease, thereby potentially altering the added value of the test. Moreover, single test research is not according to clinical practice, where a diagnosis is established after multiple tests performed in a hierarchical way, starting with simple, non-invasive and inexpensive tests, such as signs and symptoms [26]. Therefore a clinical score based on history and physical examination is included in our diagnostic model and the added value of the H-FABP bedside test will be calculated.

Previous studies with H-FABP in the hospital-setting showed the potential value of this novel cardiac biomarker in assessing patients suspected of ACS. Since GPs are confronted with patients suspected of an acute coronary syndrome at a very early stage, mostly without the availability of an ECG, the impact of a novel cardiac biomarker on the diagnostic assessment is potentially much higher in general practice than in the emergency room. To answer the question whether H-FABP has (additional) diagnostic value in the diagnosis of ACS in the primary care setting, the test needs to be studied before its introduction in that specific setting.

Application of an early biomarker potentially reduces diagnostic uncertainty in patients suspected of an ACS. On the one hand this may lead to a reduction of unnecessary hospital referrals, patient burden, hospital work load and health care costs. On the other hand, a diagnosis of ACS can be established much earlier than with troponin which may result in earlier initiation of treatment, including revascularization interventions.

To our knowledge, our study is the first to assess the (added) diagnostic value of H-FABP in patients with chest pain or other complaints suggestive of ACS in primary care.

## Competing interests

The study was supported by a grant from the Netherlands Organisation for Health Research and Development (ZonMw grant 945-06-009). Test panels were provided by Clindia Benelux BV, Leusden, the Netherlands. All authors are fully independent from the funder.

## Authors' contributions

All authors contributed to the conception and design of the study. MHEBS, FHR and GJMGH drafted the manuscript. FHR, EGM and ACB also participated in the outcome panel meetings. All authors read and approved the final draft of the manuscript.

## Pre-publication history

The pre-publication history for this paper can be accessed here:


